# Physiological and Evolutionary Changes in a Biological Control Agent During Prey Shifts Over Several Generations

**DOI:** 10.3389/fphys.2018.00971

**Published:** 2018-07-19

**Authors:** Mei-Lan Chen, Tao Wang, Yu-Hao Huang, Bo-Yuan Qiu, Hao-Sen Li, Hong Pang

**Affiliations:** State Key Laboratory of Biocontrol, Ecology and Evolution, School of Life Sciences, Sun Yat-sen University, Guangzhou, China

**Keywords:** *Cryptolaemus montrouzieri*, biological control, non-target effects, prey shifts, life history traits, gene expression, genotype frequency

## Abstract

Biological control agents usually suffer from a shortage of target prey or hosts in their post-release stage. Some predatory agents turn to attacking other prey organisms, which may induce physiological and evolutionary changes. In this study, we investigated life history traits, gene expression and genotype frequency in the predatory ladybird beetle *Cryptolaemus montrouzieri* during experimental prey shifts. *C. montrouzieri* were either continuously fed on aphids *Megoura japonica* as an alternative prey for four generations or were shifted back to the initial prey mealybugs *Planococcus citri* in each generation. In general, the utilization of aphids resulted in reduced performance and severe physiological adjustments, indicated by significant changes in development and fecundity traits and a large number of differentially expressed genes between the two offering setup prey treatments. Within the aphid-fed lines, performance regarding the developmental time, the adult weight and the survival rate recovered to some level in subsequent generations, possibly as a result of adaptive evolution. In particular, we found that a shift back to mealybugs caused a gradual increase in fecundity. Accordingly, a genotype of the fecundity-related gene *vitellogenin*, of which there were several minor alleles in the initial population, became the main genotype within four generations. The present study explored the short-term experimental evolution of a so-call specialist predator under prey shift conditions. This potential rapid adaptation of biological control agents to novel prey will increase environmental risks associated with non-target effects.

## Introduction

In biological control programs, natural enemies of pests are translocated, mass-reared and introduced as biological control agents. Following the release of these agents in their new ranges, their populations are subjected to new environmental conditions, including novel potential prey or hosts. In the post-release stage, with the decline of pest populations, the released biological control agents will suffer from a shortage of target prey or hosts. Some will therefore attack non-target organisms, which sustain the populations of agents and sometimes expand the range of controlled pests. On the other hand, there is growing concern about the side-effects of their prey or host expansion (Louda et al., 2003; van Lenteren et al., 2003; De Clercq et al., 2011). Empirical evidence has shown that the non-target effects of biological control agents have threatened complex biological communities and led to a negative impact on local environments [e.g., *Harmonia axyridis* (Koch, 2003) in Europe and North America and *Cactoblastis cactorum* (Zimmermann et al., 2004), and *Compsilura concinnata* (Elkinton and Boettner, 2012) in North America]. Tests of host or prey range are therefore among the key procedures currently used to evaluate the potential environmental risks of an introduced agent in the pre-release stage (van Lenteren et al., 2003). In this context, the release of most generalists is now restricted, while specialists are still widely used as environmentally safe agents.

In the context of evolution, utilization of alternative food resources may act as an evolutionary driver in insects. The physiological systems of insects are confronted with various chemical components from their novel diets. Consequently, dietary shifts may impose new selection pressures, driving early physiological plasticity and subsequent evolutionary changes (Vogel and Musser, 2014; Hoang et al., 2015). Thus, it is hypothetically possible that specialists are still capable of utilizing non-target prey or hosts through an adaptation process. However, there is still little evidence of adaptive prey or host expansion to non-target organisms among specialist biological control agents (Wright and Bennett, 2017).

Numerous lines of evidence have supported the idea that herbivorous arthropods evolve in association with their host plants. On a macro-evolutionary scale, the reconstructed phylogenetic trees of such species provide histories of insect-plant co-evolution [e.g., in butterflies (Janz et al., 2006), weevils (McKenna et al., 2009), and mites (Li et al., 2016a)]. On a micro-evolutionary scale, some host-associated populations are deeply divergent according to genetic marker analyses, indicating rapid evolutionary changes caused by host shifts [e.g., in thrips (Brunner et al., 2004), wasps (Stireman et al., 2005), and mites (Li et al., 2014)]. The physiological changes in host shifts reflected by expression profiling have generally involved detoxification (as reviewed in Li et al., 2007; Vogel and Musser, 2014). Accordingly, some detoxification-related genes have evolved during host adaptation [e.g., *glutathione S-transferase* in fruit flies (Matzkin, 2008), *nitrile-specifier protein* in butterflies (Wheat et al., 2007) and *cytochrome P450* in fruit flies (Bono et al., 2008), and aphids (Bass et al., 2013)]. Thus far, studies on the evolutionary changes caused by dietary shifts have been restricted to herbivorous arthropods. In contrast, the evolution of carnivorous arthropods due to dietary shifts has seldom been considered (Grenier and De Clercq, 2003). After being sustained by artificial diets over a long period, carnivorous biological control might lose the ability to capture and kill live prey, although this conjecture has not been supported by any published research (Riddick, 2008). Thus, the evolutionary potential and patterns of carnivorous arthropods during prey shifts remain largely unclear, which hampers the environmental risk assessment of specialist predators used in biological control programs.

*Cryptolaemus montrouzieri* (well known as the mealybug destroyer) is native to Australia, and is now used worldwide as a specialist predator of mealybugs in biological control (Ślipiński, 2007). It can feed on quite a broad range of mealybug species (Kairo et al., 2013). And no significant change in developmental traits was observed in the use of different mealybug species (Qin et al., 2014). However, it can also feed on aphids, whiteflies and the eggs of moths or other ladybirds under laboratory conditions (Maes et al., 2014), suggesting potential non-target effects in its field use. Some non-target diets can sustain a complete life history (e.g., *Ephestia kuehniella* eggs) but are overall less suitable for survival, development, and reproduction (Maes et al., 2014), suggesting that a further adaptation process occurs when these diets are used continuously. Moreover, the macro-evolutionary pattern of the ladybird family Coccinellidae based on molecular phylogeny supports the idea that dietary shifts have played an important role in species diversification (Giorgi et al., 2009; Escalona et al., 2017). To predict the potential for and consequences of non-target effects of *C. montrouzieri*, we previously examined its response to the novel prey species *Megoura japonica*, a common aphid pest in China, and detected reduced performance and expression of genes related to biochemical transport, metabolism, and detoxification (Li et al., 2016b). However, the question of whether these physiological changes in response to alternative prey were simply plastic or had further consequences remained unsolved.

In the present study, we test whether evolution occurs associated with prey shift of a predatory biological control agent. We used an experimental evolutionary approach (Kuhnle and Muller, 2011; Muller et al., 2017) to test the potential consequences of prey shifts in the use of *C. montrouzieri* for biological control. An initial population was either continuously fed alternative prey for four generations or shifted back to the initial prey in each generation. Life history traits and gene expression were investigated in each generation/prey treatment. In particular, due to pronounced changes in fecundity traits, we also examined the changes in the genotype frequencies of fecundity-related genes across generations.

## Materials and Methods

### Insect Rearing

The ladybird *C. montrouzieri* used in this study were obtained from a population at Sun Yat-sen University, Guangzhou, China, that has been maintained under laboratory conditions with mealybugs as prey for more than 10 years. The initial prey of *C. montrouzieri*, the citrus mealybug *Planococcus citri*, was maintained on fruits of the pumpkin *Cucurbita moschata*. The alternative prey aphid *M. japonica* was maintained on plants of the horse bean *Vicia faba*. In the experimental stages, all insects and plants were kept in climate chambers set at 25 ± 1°C with 75 ± 5% relative humidity (RH) and a 14:10 (L:D) h photoperiod.

### Experimental Prey Shifts

**FIGURE 1:**
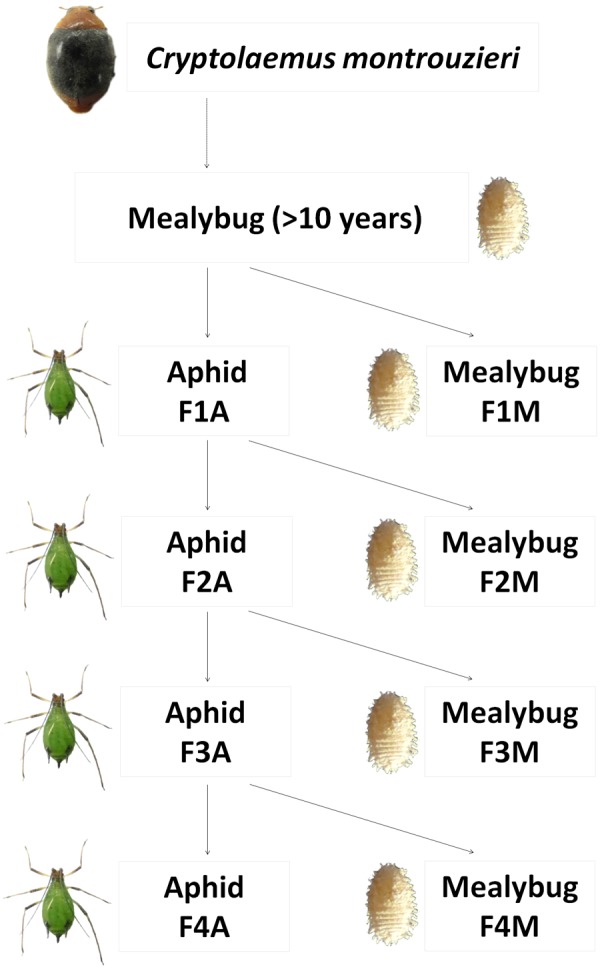
Scheme of prey shifts in the evolutionary experimental approach applied in this study. *Cryptolaemus montrouzieri* ladybirds were reared on *Planococcus citri* mealybugs for more than 10 years (∼12 generations per year) and then allocated to two lines, either maintained on the alternative prey *Megoura japonica* aphids (F1A) or further reared on mealybugs (F1M). Freshly hatched larvae from F1A eggs were either shifted to mealybugs (F2M) or further reared on aphids (F2A). The lines F3M, F3A, F4M, and F4A were generated in a similar way.

A prey shift during the biological control release of *C. montrouzieri* was simulated under laboratory conditions as shown in **Figure 1**. In detail, first-instar larva from the original population of *C. montrouzieri* (<24 h old) were distributed into two different lines, which were then either maintained on mealybugs (designated “F1M”) or shifted to aphids (first generation reared on aphids, designated “F1A”). Freshly hatched larvae from F1A eggs were again randomly distributed on the initial prey mealybugs (F2M) or reared on aphids (F2A). The F3M, F3A, F4M, and F4A lines were generated similarly, resulting in a total of eight lines.

### Comparison of Life History Traits

The life history traits of the eight lines were investigated during the experimental prey shifts. Developmental traits including the survival rate and the development time of larvae as well as the adult weight and the sex ratio were investigated with 85 individuals for F1A, 135 for F2A, 93 for F3A, 96 for F4A, 91 for F1M, 63 for F2M, 83 for F3M, and 90 for F4M. Freshly hatched larvae were placed individually in plastic Petri dishes (3.5 cm diameter, 1.2 cm height), and their prey were offered *ad libitum* and replenished daily. The survival and the developmental time of the ladybird larvae were monitored daily. Newly emerged adults (no food or water was provided) were weighed and sexed based on the color of forelegs (Babu and Azam, 1987).

Fecundity traits, including the pre-oviposition time and the number of deposited eggs, were also surveyed, with 11 adult pairs for F1A, 10 for F2A, 11 for F3A, 14 for F4A, 12 for F1M, 12 for F2M, 14 for F3M, and 14 for F4M. Newly emerged males and females were paired in plastic Petri dishes (5 cm diameter, 2 cm height) and continuously fed by aphids or mealybugs. A piece of cotton (∼1 cm × 1 cm) was provided as an oviposition substrate and was checked daily for eggs to determine the pre-oviposition period. Once the first egg was laid, the substrates were replaced, and the number of eggs was monitored three times a week for a total period of 30 days.

Variations of all life history traits were analyzed using SPSS 20.0 (SPSS Inc.). The Kolmogorov–Smirnov test indicated that the adult weight of both males and females was normally distributed and was therefore analyzed using one-way analysis of variance (ANOVA). Because the Levene test indicated homoscedasticity, the means were separated using Tukey tests. According to the Kolmogorov–Smirnov test, the developmental time, the pre-oviposition time and the number of deposited eggs were not normally distributed. Therefore, we used the non-parametric Kruskal–Wallis *H* test, followed by the Mann–Whiney *U* test. The significance level of all tests was set at *p* < 0.05.

### Comparison of Gene Expression

Genome-wide expression profiling based on transcriptome sequencing was performed to screen for regulation of expression in response to the experimental prey shifts. Two females from each line were collected on the 30th day of the oviposition period for transcriptome sequencing (designated F1A1, F1A2, F2A1, F2A2, F3A1, F3A2, F4A1, F4A2, F1M1, F1M2, F2M1, F2M2, F3M1, F3M2, F4M1, and F4M2). RNA was extracted from the whole individual after 12 h of starvation. RNA extraction, RNA-seq analysis, data quality control, *de novo* assembly and unigene annotation followed Li et al. (2016b). Specifically, we used the FPKM (fragments per kilobase of transcript per million mapped reads) method to normalize gene expression (Trapnell et al., 2010). We removed the genes showing low expression, with an FPKM < 1, from further analysis. The regulation of gene expression in each pair of lines was tested using DESeq (Anders and Huber, 2010), employing a fold change >2 or <0.5 and a false discovery rate (FDR) < 0.05 were the criteria for defining differentially expressed genes (DEGs). To further characterize these DEGs, the number of DEGs in each pair of lines and their distribution according to EuKaryotic Orthologous Groups (KOG) classification were calculated. To visualize the expression profiles, heatmaps and clustering of the normalized expression of DEGs were generated using R (R Development Core Team, 2013).

Since we focused on fecundity changes under prey shifts in this study, potential fecundity-related genes of *C. montrouzieri* were selected based on a comprehensive literature review and a recent insect fecundity study (Gilbert et al., 2005; Sun et al., 2015). A total of 10 selected genes were detected in the transcriptome data obtained in this study: *vitellogenin* (*Vg*), *vitellogenin receptor* (*VgR*), *3-hydroxy-3-methylglutaryl-CoA reductase* (*HMGCR*), *angiotensin converting enzyme gene* (*ACE*), *Fizzy, sex-lethal* (*Sxl*), *heat shock protein 70* (*HSP70*), *Hunchback, heat shock protein 90* (*HSP90*), and *bicaudal D* (*BicD*). The detail functions of these genes can be found in Sun et al. (2015). Heatmaps of the normalized expression of these genes were generated.

### Allele Frequencies of Fecundity-Related Genes

The transcriptome analysis also enabled us to initially detect changes in allele frequencies of unigenes. First, single nucleotide polymorphisms (SNPs) of unigenes in the transcriptome were screened using GATK (McKenna et al., 2010). Then, the transcriptome data of four individuals within each generation were grouped. For example, F1A1, F1A2, F1M1, and F1M2 were grouped as F1. The allele frequencies and coding changes in the SNPs of 10 fecundity-related genes among F1–F4 were counted.

We further validated the pattern of allele frequency changes in *Vg*, a fecundity-related gene, in a larger sample. We partially repeated the experiment using the prey shift system described above, where a new subset of the original population was continuously fed with the alternative prey, *M. japonica* aphids, for five generations to generate new F1–F5 populations. Two pairs of primers were designed and used to amplify two *Vg* fragments that included all SNP loci (Supplementary Table 1). Twenty-eight to thirty individuals from each generation were randomly selected. DNA was then extracted, and polymerase chain reaction (PCR) and sequencing of the products were then performed as described in Li et al. (2015). The allele frequencies in the SNPs were subsequently counted, and the haplotype networks of the two PCR fragments were drawn in Network 5.0.0.3 using a median-joining method (Bandelt et al., 1999).

## Results

### Life History Traits

**FIGURE 2:**
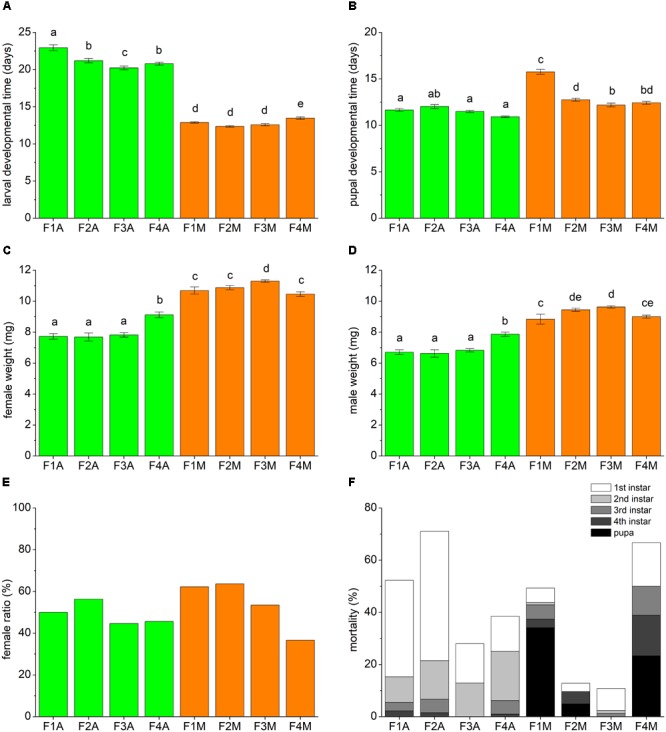
Developmental traits of each line, including **(A)** the larval developmental time, **(B)** the pupal developmental time, **(C)** the female weight, **(D)** the male weight, **(E)** the female ratio, and **(F)** the mortality rate. Approximately 100 freshly hatched larvae of each line were tested. Bars (mean ± standard error of mean) with the same letter are not significantly different (*p* ≥ 0.05).

The developmental traits of each line, including the development time, the adult weight, the female ratio and the mortality, are shown in **Figure 2**. In comparison with the mealybug-fed lines, the aphid-fed lines always exhibited significantly longer larval developmental times (**Figure 2A**) and lower weights of adult males and females (**Figures 2C,D**). When four generations were continuously fed aphids, the larval developmental time decreased from F2A onward (**Figure 2A**), and the adult weight increased in F4A (**Figures 2C,D**). The overall mortality among developmental stages was increased in F2A compared with F1A but decreased greatly in F3A and F4A (**Figure 2F**). The shift back to mealybugs led to an increase in larval developmental time in F4M (**Figure 2A**) and a decrease in pupal developmental time from F2M onward (**Figure 2B**). In addition, it appeared that there was no strong effect of the prey shift on the female ratio (**Figure 2E**).

**FIGURE 3:**
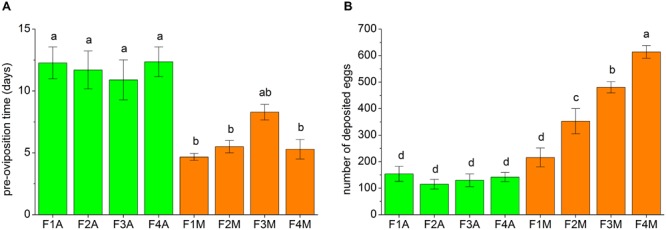
Fecundity traits of each line, including **(A)** the pre-oviposition time and **(B)** the number of deposited eggs within the first 30 days of the oviposition period. 10–14 pairs of adults from each line were tested. Bars (mean ± standard error of mean) with the same letter are not significantly different (*p* ≥ 0.05).

The fecundity traits of each line, including the pre-oviposition time and the number of eggs deposited within 30 days, are shown in **Figure 3**. The pre-oviposition time of the aphid-fed lines was usually significantly longer than that of the mealybug-fed lines (**Figure 3A**). However, the number of deposited eggs in F1M was not significantly higher than that in F1A (**Figure 3B**). The number of deposited eggs did not significantly change between generations in the aphid-fed lines but gradually increased from the first to the fourth generation in the lines shifted back to mealybugs (**Figure 3B**).

### Gene Expression

The transcriptomes of all eight lines and 16 female individuals were sequenced. Each of the sequenced samples exhibited 23–34 million high-quality reads, comprised of 5–11 billion base pairs (bp). A total of 162 genes were considered DEGs across the treatments experiment-wide. The KOG classification of these DEGs showed that most were distributed among carbohydrate, amino acid, lipid transport and metabolism, energy production and conversion, and signal transduction mechanisms (Supplementary Figure 1). Among these DEGs identified experiment-wide, an average of 19.33 DEGs were identified between pairs of aphid-fed lines, 32.50 between pairs of mealybug-fed lines, and 47.38 between pairs of aphid- and mealybug-fed lines (Supplementary Table 2). The heatmaps of the expression of all DEGs showed that their expression depended on not only their prey species but also their generation (Supplementary Figure 2).

**FIGURE 4:**
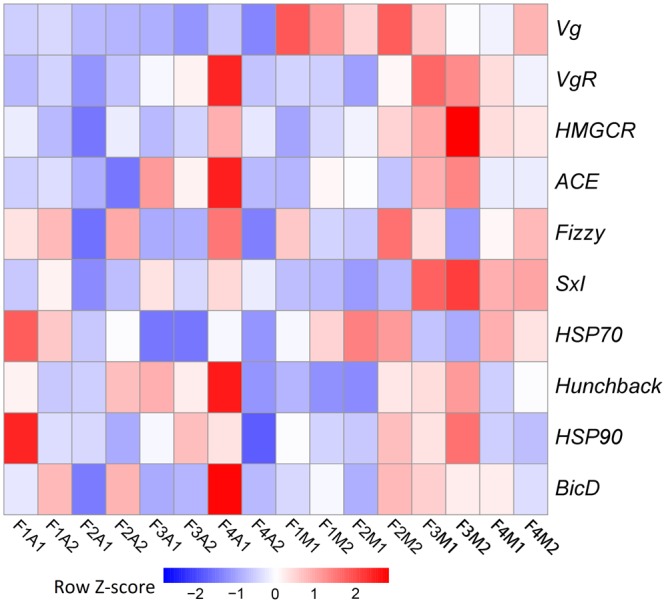
Heatmaps based on the normalized expression of 10 selected fecundity-related genes in each line.

Due to the pronounced change observed in fecundity, we further focused on potential genetic changes associated with fecundity under prey shifts. Among the 10 selected fecundity-related genes, none was a DEG. *Vg* was the only gene that was always expressed at a lower level in aphid-fed lines than in mealybug-fed lines (**Figure 4**).

### Allele Frequencies of Fecundity-Related Genes

A total of 93 SNPs in fecundity-related genes were detected in the transcriptome data (Supplementary Table 3). Among these SNPs, 4/7 were involved in non-synonymous changes in *Vg*, 9/22 in *VgR*, 0/9 in *HMGCR*, 1/17 in *ACE*, 2/11 in *Fizzy*, 0/6 in *Sxl*, 0/11 in *HSP70*, 0/5 in *HSP90*, and 1/7 in *DisC*. Among these non-synonymous SNPs, those in *Vg* exhibited the greatest allele frequency changes from F1 (or the initial population) to F4, ranging from 0.375 to 1 (**Table 1**).

**Table 1:** Single nucleotide polymorphisms (SNPs) in *vitellogenin* (*Vg*) and their allele frequencies from F1 to F4 (or F5).

Position	Allele	Translate	Data	F1	F2	F3	F4	F5
*Vg*_679_	G > T	K > N	Transcriptome	0.375	0.875	1	1	–
			Validation	0.133	0.03	0.1	0.786	1
*Vg*_1719_	A > G	P > S	Transcriptome	0.375	0.875	1	1	–
			Validation	0.533	0.121	0.133	0.821	1
*Vg*_1919_	C > T	K > R	Transcriptome	0.375	0.875	1	1	–
			Validation	0.37	0.133	0.1	0.75	1
*Vg*_1920_	G > T	K > R	Transcriptome	0.375	0.875	1	1	–
			Validation	0.185	0.033	0.1	0.679	1

The repeated rearing scheme and Sanger sequencing-based validation showed that the frequencies of these four minor alleles in *Vg* involving non-synonymous changes ranged from 0.133 to 0.533 in F1 (or the initial population). The frequencies of these alleles did not change greatly in F2 and F3 but increased to 0.679–0.821 in F4, and these alleles were the only alleles detected in F5 (**Table 1**). Additionally, the haplotype network of the two PCR fragments suggested that a rare haplotype in F1-F3 became the main haplotype in F4 and F5 (Supplementary Figure 3).

## Discussion

### Physiological Changes During Feeding on Non-target Prey

Diet is one of the major determinants of physiological performance. When their diet changes, both herbivores and carnivores usually require physiological adjustments to meet their nutritional requirements and cope with new toxins from their new diet (Glendinning, 2007; Vogel and Musser, 2014). In the present study, the initial prey (mealybugs) and the alternative prey (aphids) exhibited different biochemical compositions (Brown, 1975) and should present differences in nutritional value or toxins, with which *C. montrouzieri* would have to cope. Based on the evidence regarding life history traits and gene expression, we found that both the shift to aphids and the shift back to mealybugs led to severe physiological changes in *C. montrouzieri*. The shift to aphids always led to reduced performance, as inferred by the significant extension of development and pre-oviposition times and the decreases in adult weight and the number of deposited eggs. Furthermore, the whole-genome expression profiles after prey shifts mainly showed modulation of the expression of genes related to biochemical transport and metabolism. This transcriptional plasticity is expected be a response to the change in the biochemical composition of the prey. These findings regarding physiological changes were in line with those from previous studies in which *C. montrouzieri* was switched to aphid prey for one generation (Maes et al., 2014; Li et al., 2016b).

### Short-Term Adaptation Under Prey Shifts

Because *C. montrouzieri* experienced performance reductions and physiological adjustments when feeding on aphids, we assumed that further adaptation occurred when the ladybirds were continuously maintained on this alternative prey. Several reports of experimental evolution in herbivorous arthropods support the idea that dietary shifts impose strong selection pressure, forcing herbivores to evolve within several generations (Sezer and Butlin, 1998; Warbrick-Smith et al., 2006; Kuhnle and Muller, 2011). In this study, variation of life history traits and gene expression was also detected in lines within the same prey treatment. Within the aphid-fed lines, performance recovered to a certain degree in subsequent generations, as inferred by the larval developmental time (recovered from F2), the female and male weight (recovered from F4) and the mortality (recovered from F3). It appeared that the physiological plasticity of the initial population was already sufficient for utilization of the alternative prey. However, performance under this adverse condition was reduced, and positive selection is therefore expected to further alter performance in the direction of the optimum (Crispo, 2007; Hoang et al., 2015). In addition to positive selection, this prey adaptation might be a result of genetic drift or a maternal effect.

### Change in Fecundity and Its Potential Genetic Basis

In this study, the change in fecundity was the most pronounced physiological change observed experiment-wide. In the lines shifted back to mealybugs from aphids, the younger generations always exhibited a significantly greater number of deposited eggs than the older generations. To explore the potential genetic basis of this change in fecundity, we first selected candidate genes from 10 fecundity-related genes based on the regulation of their expression under prey shifts. Among these candidate genes, only the expression of *Vg* in females was consistently altered in response to prey type. Similarly, in a previous study, we found that when *C. montrouzieri* was switched to aphids, *Vg* was significantly downregulated, while other fecundity-related genes were not differentially expressed (Li et al., 2016b). *Vg* encodes the major egg yolk protein precursor in insects, and its expression generally corresponds to fecundity in females (Tufail et al., 2014). In this study, however, the increase in fecundity was not based on increased expression of *Vg*; rather, it is expected to be a consequence of genetic changes.

Therefore, we further tested the potential genetic changes in fecundity-related genes in response to this change in fecundity. Again, *Vg* exhibited the greatest change in allele frequencies in non-synonymous SNPs in this prey shift experiment. In this process, a genotype that contained several minor alleles in the initial population became the main genotype in F2 and the only genotype in F3. Replication of this prey shift experiment showed that this minor genotype became the main genotype in F4 and the only genotype in F5. Based on the results of these two replicate experiments, we suggested that positive selection, not random genetic drift, led to this change in genotype frequency.

Increased fecundity is considered to be adaptative (Stearns and Hoekstra, 2003). It has been reported that the fecundity of insects can be affected by several mutations in specific genes (Sun et al., 2015), and a host shift of insects may lead to the evolution of genes related to reproduction (Guillen et al., 2014). In this study, we explored the downregulation of *Vg* during the utilization of alternative prey as well as the relationship between the *Vg* genotype frequency and fecundity in lines that were shifted back to the initial prey. However, the changes in *Vg* genotype frequency neither increased the fecundity of the aphid-fed lines nor affected *Vg* expression to a significant level. We suggest that this lack of change was due to the limitation of fecundity by insufficient nutritional conditions associated with the alternative prey, even though a selected genotype presents the potential for higher fecundity. On the other hand, the selection pressure exerted by the alternative prey might be exerted directly on *Vg* or indirectly on other genes linked to *Vg*.

### Conclusion and Implications for Biological Control

This study explored the short-term physiological and evolutionary changes in a so-call specialist biological control agent during experimental prey shifts over several generations. For better explaining this prey shift adaptation, other factors such as behavior and gut microbiota needed to be considered in the further. This is the first report to address the experimental evolution of carnivorous arthropods and biological control agents under prey shifts. This potential rapid adaptation of biological control agents to novel prey will increase environmental risks associated with non-target effects. Hence, the current environmental risk assessment strategy based on the host ranges of biological control agents (van Lenteren et al., 2003) should also include evolutionary considerations.

## Data Availability Statement

All raw sequence data generated and analyzed for this study can be found in the NCBI Short Read Archive (SRA) under BioProject ID PRJNA449583 (BioSample ID: SAMN08912452–SAMN08912467).

## Author Contributions

M-LC, Y-HH, and B-YQ performed the experiments. M-LC and H-SL analyzed the data. M-LC, TW, H-SL, and HP wrote and revised the manuscript.

## Conflict of Interest Statement

The authors declare that the research was conducted in the absence of any commercial or financial relationships that could be construed as a potential conflict of interest.
