# Supplementary material for: Physiological and Evolutionary Changes in a Biological Control Agent During Prey Shifts Over Several Generations
Source: Front Physiol. 2018 Jul 19;9:971. doi: 10.3389/fphys.2018.00971 (PMC6060241; doi:10.3389/fphys.2018.00971)
Supplement: Supplementary file 1 [file Presentation_1.PDF]

Supplementary Material

Physiological and evolutionary changes in a biological control agent during prey shifts over several generations

Mei-Lan Chen[#], Tao Wang[#], Yu-Hao Huang, Bo-Yuan Qiu, Hao-Sen Li[*], Hong Pang[*]

*** Correspondence:** Hao-Sen Li: lihaosen3@mail.sysu.edu.cn; Hong Pang: lsshpang@mail.sysu.edu.cn

1 Supplementary Figures and Tables

1.1 Supplementary Figures

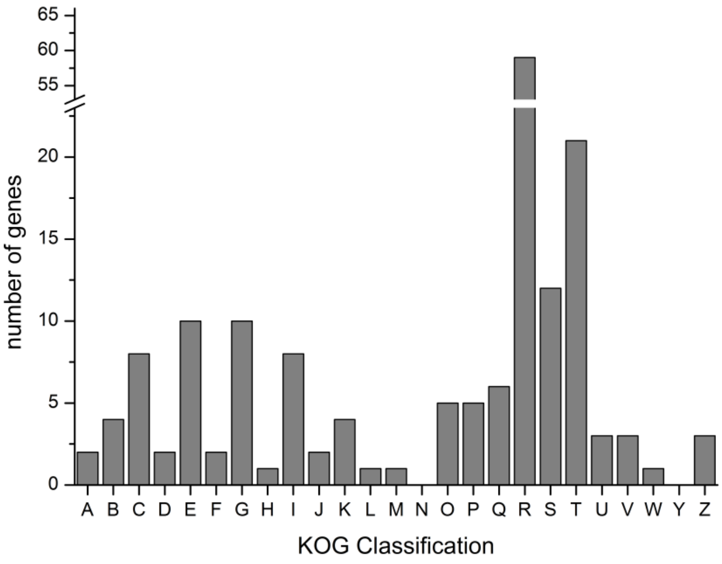

Supplementary Figure 1. EuKaryotic Orthologous Groups (KOG) classification of differentially expressed genes (DEGs). A: RNA processing and modification, B: chromatin structure and dynamics, C: energy production and conversion, D: cell cycle control, cell division, chromosome partitioning, E: amino acid transport and metabolism, F: nucleotide transport and metabolism, G: carbohydrate transport and metabolism, H: coenzyme transport and metabolism, I: lipid transport and metabolism, J: translation, ribosomal structure and biogenesis, K: transcription, L: replication, recombination and repair, M: cell wall/membrane/envelope biogenesis, N: cell motility, O: posttranslational modification, protein turnover, chaperones, P: inorganic ion transport and metabolism, Q: secondary metabolites biosynthesis, transport and catabolism, R: general function prediction only, S: function unknown, T: signal transduction mechanisms, U: intracellular trafficking, secretion, and vesicular transport, V: defense mechanisms, W: extracellular structures, Y: nuclear structure, Z: cytoskeleton.

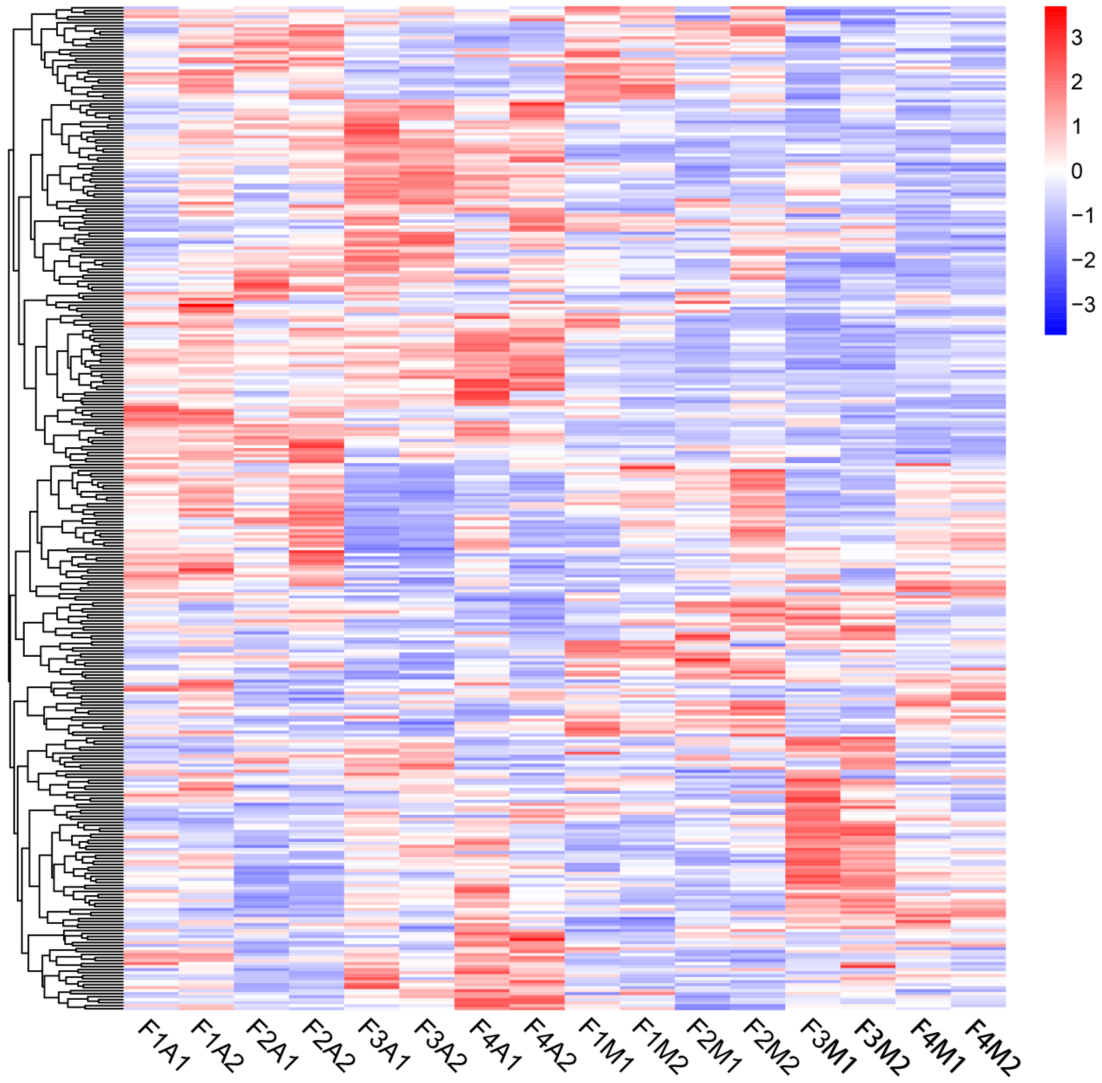

Supplementary Figure 2. Heatmaps and clustering base on the normalized expression of all differentially expressed genes (DEGs) experiment-wide.

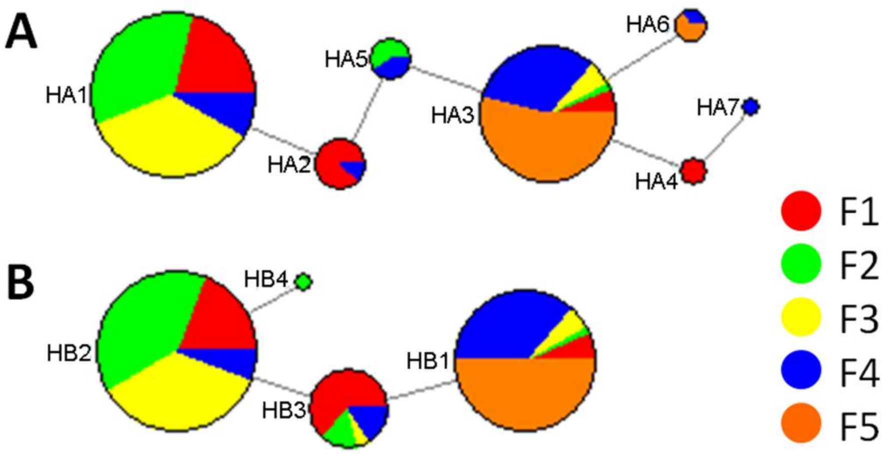

Supplementary Figure 3. Haplotype networks of individuals from F1 to F5 based on two PCR fragments in the validation system.

1.2 Supplementary Tables

Supplementary Table 1. primers used to amplify *vitellogenin* (*Vg*).

region	name	sequence
part A	vite3465f	ATGCCACAACAATCTCAGAACAAGA
part A	vite3916r	AACTCGATAGCTGCGGTAGAAGTAAG
part B	vite4509f	ACGGTCCTTCTTCAATATGTTCCTC
part B	vite4941r	CGAAGTTTCCGCAAGCTCCTC

Supplementary Table 2. Number of differentially expressed genes (DEGs) in each pair of lines.

	F1A	F2A	F3A	F4A	F1M	F2M	F3M	F4M
F1A								
F2A	5							
F3A	22	35						
F4A	14	36	4					
F1M	21	17	53	45				
F2M	22	11	87	81	12			
F3M	53	93	48	65	30	78		
F4M	15	31	74	42	21	15	39	

Supplementary Table 3. Information on single nucleotide polymorphisms (SNPs) in ten selected fecundity-related genes, including positions, alleles, coding changes and allele frequencies in four generations.

gene	position	allele	coding	F1	F2	F3	F4
Vg	679	G>T	K>N	0.375	0.875	1	1
Vg	733	A>G	synonymous	0.375	0.875	1	1
Vg	1719	A>G	P>S	0.375	0.875	1	1
Vg	1919	C>T	K>R	0.375	0.875	1	1
Vg	1920	G>T	K>R	0.375	0.875	1	1
Vg	4846	A>G	synonymous	0.375	0.875	1	1
Vg	5221	A>G	synonymous	0.375	0.875	1	1
VgR	1388	C>T	synonymous	0.75	1	1	1
VgR	1421	G>A	synonymous	0.75	1	1	1
VgR	1550	T>A	synonymous	0.75	1	1	1
VgR	2322	G>A	V>L	0.875	0.875	0.625	0.875
VgR	2760	A>T	T>S	0.625	0.875	0.625	0.875
VgR	2907	A>G	K>E	0.625	0.875	0.625	0.875
VgR	2939	C>T	synonymous	0.625	0.875	0.625	0.875
VgR	2969	T>C	synonymous	0.625	0.875	0.625	0.875
VgR	3286	G>A	R>Q	0.625	0.875	0.625	0.875
VgR	3386	G>T	synonymous	0.625	0.875	0.625	0.875
VgR	3630	G>A	E>K	0.875	0.875	0.625	0.875
VgR	3665	A>G	synonymous	0.75	1	1	1

VgR	3827	G>C	synonymous	0.625	0.875	0.625	0.875
VgR	3840	A>C	Q>K	0.625	0.875	0.625	0.875
VgR	3863	C>A	synonymous	0.875	0.875	0.625	0.875
VgR	3963	A>G	N>D	0.625	0.875	0.625	0.875
VgR	4061	G>A	synonymous	0.75	1	1	1
VgR	4373	T>G	synonymous	0.625	0.875	0.625	0.875
VgR	5075	C>A	synonymous	0.625	0.875	0.625	0.875
VgR	5124	T>A	S>T	0.625	0.875	0.625	0.875
VgR	5129	A>G	synonymous	0.625	0.875	0.625	0.875
VgR	5181	G>A	A>T	0.625	0.875	0.625	0.875
HMGCR	1785	T>C	synonymous	0.625	0.625	1	1
HMGCR	1830	T>G	synonymous	0.625	0.625	1	1
HMGCR	1878	T>A	synonymous	0.875	0.75	1	1
HMGCR	1953	T>C	synonymous	0.625	0.625	1	1
HMGCR	1954	C>T	synonymous	0.625	0.625	1	1
HMGCR	1992	T>C	synonymous	0.625	0.625	1	1
HMGCR	2016	A>G	synonymous	0.625	0.625	1	1
HMGCR	2127	G>A	synonymous	0.875	0.875	1	1
HMGCR	2226	T>C	synonymous	0.875	0.875	1	1
ACE	310	G>T	synonymous	0.875	1	1	1
ACE	491	C>T	synonymous	0.875	1	1	1
ACE	596	T>A	synonymous	0.5	0.5	0.75	1
ACE	652	G>A	synonymous	0.875	1	1	1
ACE	767	A>G	synonymous	0.875	1	1	1
ACE	929	T>C	synonymous	0.875	1	1	1
ACE	1058	T>C	synonymous	0.375	0.5	0.75	1
ACE	1106	A>C	synonymous	0.5	0.5	0.75	1
ACE	1196	T>G	synonymous	0.5	0.5	0.75	1
ACE	1226	A>G	synonymous	0.875	1	1	1
ACE	1304	A>T	synonymous	0.875	1	1	1
ACE	1661	A>C	synonymous	0.875	1	1	1
ACE	1762	A>C	synonymous	0.875	1	1	1
ACE	1832	C>T	synonymous	0.875	1	1	1
ACE	1859	A>C	S>A	0.875	1	1	1
ACE	2021	T>A	synonymous	0.875	1	1	1
ACE	2054	T>G	synonymous	0.875	1	1	1
Fizzy	1344	G>A	synonymous	0.5	0.5	0.125	1
Fizzy	1515	C>T	I>V	0.5	0.625	0.875	0
Fizzy	1893	A>G	synonymous	0.5	0.625	0.875	0
Fizzy	2057	C>T	synonymous	0.5	0.5	0.5	0.5
Fizzy	2112	A>G	synonymous	0.5	0.5	0.125	1
Fizzy	2301	A>G	synonymous	0.5	0.5	0.125	1
Fizzy	2493	T>A	synonymous	0.5	0.5	0.125	1
Fizzy	2619	C>T	R>G	0.5	0.5	0.125	1
Fizzy	2748	T>A	synonymous	0.5	0.5	0.125	1
Sxl	128	T>C	synonymous	0.375	0.625	0.75	0.625
Sxl	410	T>C	synonymous	0.375	0.625	0.75	0.625

Sxl	506	G>T	synonymous	0.375	0.625	0.75	0.625
Sxl	554	T>A	synonymous	0.375	0.625	0.75	0.625
Sxl	596	C>A	synonymous	0.375	0.625	0.75	0.625
Sxl	737	C>T	synonymous	0.375	0.625	0.75	0.625
HSP70	590	G>A	synonymous	0.5	0.75	0.25	0.875
HSP70	935	A>C	synonymous	0.875	1	1	0.125
HSP70	938	T>C	synonymous	0.625	0.875	0.375	0.875
HSP70	1082	A>G	synonymous	0.625	0.25	0.375	0.875
HSP70	1403	C>T	synonymous	0.875	0.875	0.875	1
HSP70	1457	T>C	synonymous	0.875	0.875	0.875	1
HSP70	1463	G>A	synonymous	0.5	0.75	0.25	0.875
HSP70	1571	G>A	synonymous	0.875	0.875	0.875	1
HSP70	1631	G>T	synonymous	0.875	0.25	0.875	1
HSP70	2180	G>A	synonymous	0.5	0.125	0.625	1
HSP70	2186	A>G	synonymous	0.625	0.875	0.75	1
HSP90	707	G>A	synonymous	0.5	0.5	0.625	0.625
HSP90	769	A>G	synonymous	0.5	0.5	0.625	0.625
HSP90	860	G>A	synonymous	0.5	0.5	0.625	0.625
HSP90	1334	G>A	synonymous	0.5	0.5	0.625	0.625
HSP90	1928	T>A	synonymous	0.5	0.5	0.625	0.625
BicD	4138	G>T	synonymous	0.875	0.875	0.75	1
BicD	4696	G>A	synonymous	0.5	0.5	0.75	0.875
BicD	4780	T>G	synonymous	0.5	0.5	0.75	0.875
BicD	5290	T>C	synonymous	0.5	0.5	0.75	0.875
BicD	5542	C>G	synonymous	0.375	0.375	0.5	0.875
BicD	5575	T>C	synonymous	0.5	0.5	0.75	0.875
BicD	6050	G>C	T>S	0.625	0.5	0.75	0.875
